# Pneumococcal Septic Shock Syndrome: A Deadly Condition Despite Vaccination

**DOI:** 10.7759/cureus.52255

**Published:** 2024-01-14

**Authors:** Liliana Costa, Sofia Silva, Núria Jorge, André Silva-Pinto, José-Artur Paiva

**Affiliations:** 1 Intensive Care Unit Department, Centro Hospitalar Universitário de São João, Porto, PRT; 2 Infectious Diseases Department, Centro Hospitalar Universitário de São João, Porto, PRT

**Keywords:** septic shock (ss), streptococcus pneumoniae, pneumococcal conjugate vaccine, asplenia, invasive pneumococcal disease

## Abstract

Invasive pneumococcal disease is a serious infection with an elevated case-fatality rate that can be even higher among patients with asplenia. Its impact has been blunted by the widespread use of vaccines; even recently, in 2021, two new pneumococcal conjugate vaccines emerged. The authors present a case of a 58-year-old male, splenectomised with the immunisation schedule complete, who died of invasive pneumococcal disease with a fulminant course. It is highlighted that fever in a patient with impaired splenic function is an emergency, and despite the success of immunisation in reducing pneumococcal carriage and invasive disease, serotypes continue to change. Also, the local epidemiology may help guide situations where the immunisation recommendations are dubious regarding the implementation of the new vaccines.

## Introduction

*Streptococcus pneumoniae* (*S. pneumoniae*) is an aerobic gram-positive coccus that causes a broad variety of infections. Non-invasive pneumococcal infections include bronchitis, otitis media, and sinusitis. An infection in which *S. pneumoniae* is isolated from a typically sterile body site is known as an invasive pneumococcal illness. The most frequent presentation is pneumonia, followed by bacteremia (in which no source is identified), meningitis, septic arthritis, spontaneous peritonitis, endocarditis, osteomyelitis, and soft tissue infection. It has long been one of the most relevant bacterial causes of disease in humans, but since 2000, its impact has been blunted by the widespread use of vaccines that largely prevent infection and colonisation in young children [1]. Since there have been over 90 distinct *S. pneumoniae* serovars, the research aims at developing vaccines that deliver broad immunity. Pneumococcal conjugate vaccine (PCV) and pneumococcal polysaccharide vaccine (PPSV) are the two forms of pneumococcal vaccines that are available for clinical use. Both the active components are capsular polysaccharides from pneumococcal serotypes that commonly cause invasive disease [2]. The pneumococcal vaccination is indicated for all adults 65 years of age or older, as well as those under 65 who are at risk for pneumococcal infection or severe complications, namely immunocompromised, those with long-term predisposing illnesses (such as lung disease), functional or anatomic asplenia, or a history of invasive pneumococcal disease. In Portugal, the recommended vaccines are the PPSV23 (Pneumovax 23^®^), which includes 23 partially purified capsular polysaccharide serotypes (1, 2, 3, 4, 5, 6B, 7F, 8, 9N, 9V, 10A, 11A, 12F, 14, 15B, 17F, 18C, 19A, 19F, 20, 22F, 23F, and 33F), and the 13-valent PCV13 (Prevnar 13^®^), which contains capsular polysaccharide antigens covalently linked to a nontoxic protein (covers serotypes 1, 3, 4, 5, 6A, 6B, 7F, 9V, 14, 18C, 19A, 19F, 23F) [3]. As serotypes that cause pneumococcal disease continue to change, in the summer of 2021, two new PCVs for use in adults emerged: PCV15 and PCV20 [4]. These are approved and commercialised in Portugal, although they are not yet cited in the national guidelines.

## Case presentation

**Figure 1:**
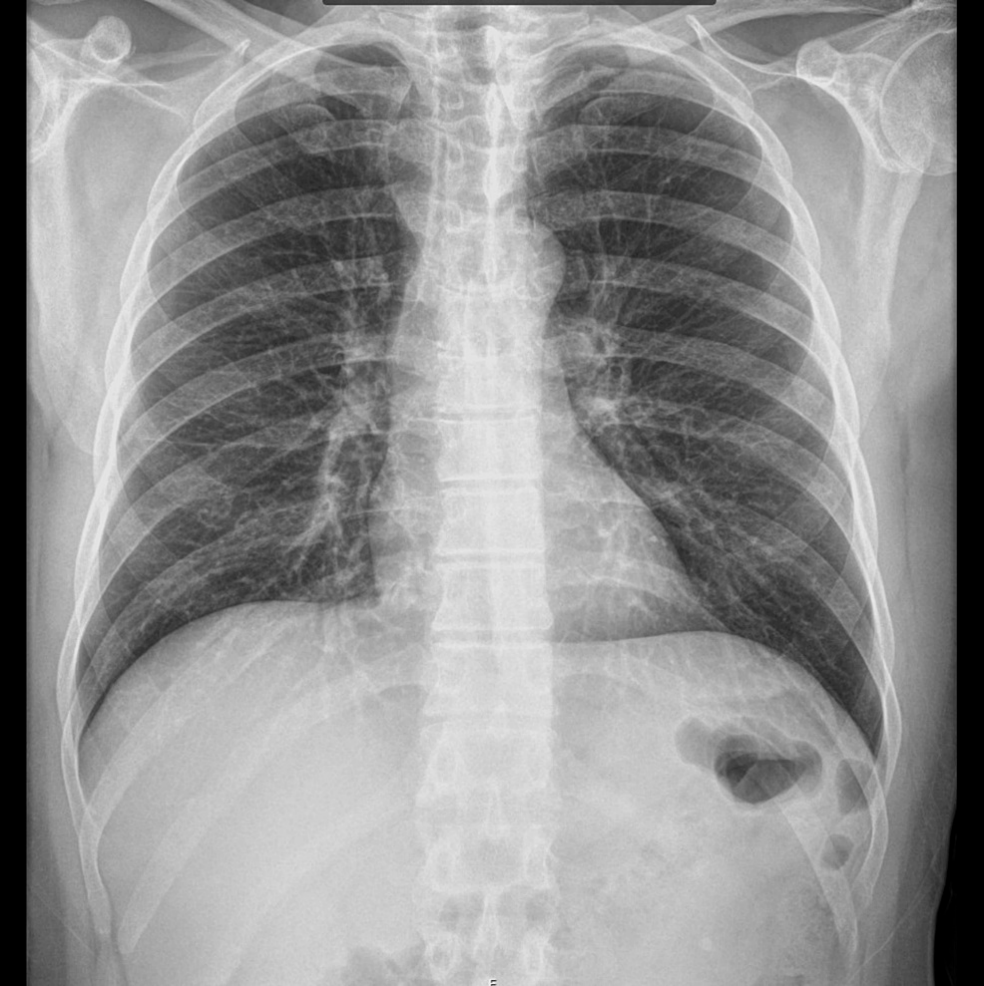
Chest X-ray on the first admission showing no significant alteration

**Figure 2:**
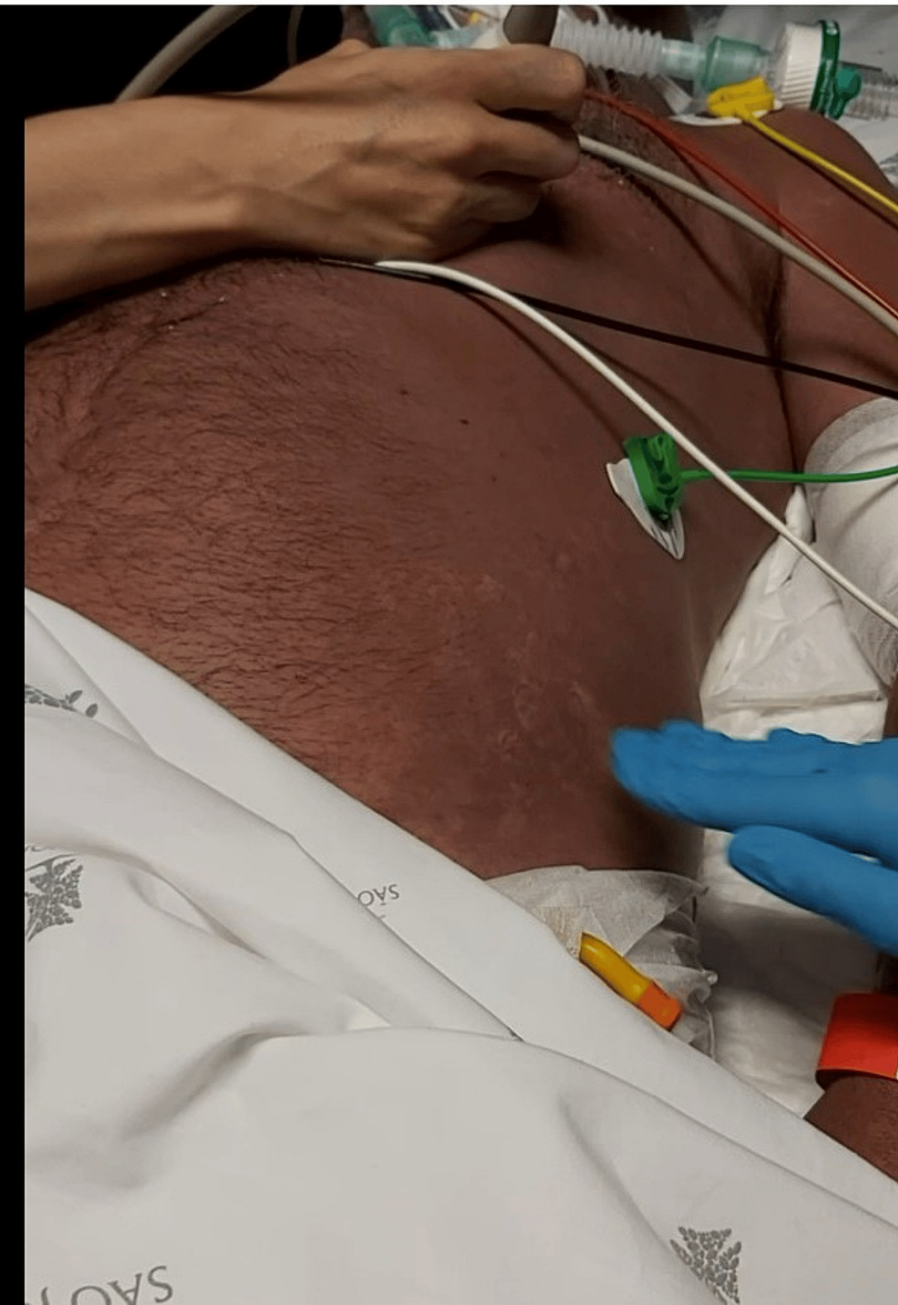
Diffuse, exuberant erythematous rash that blanches on pressure, a rare finding in streptococcal shock

**Figure 3:**
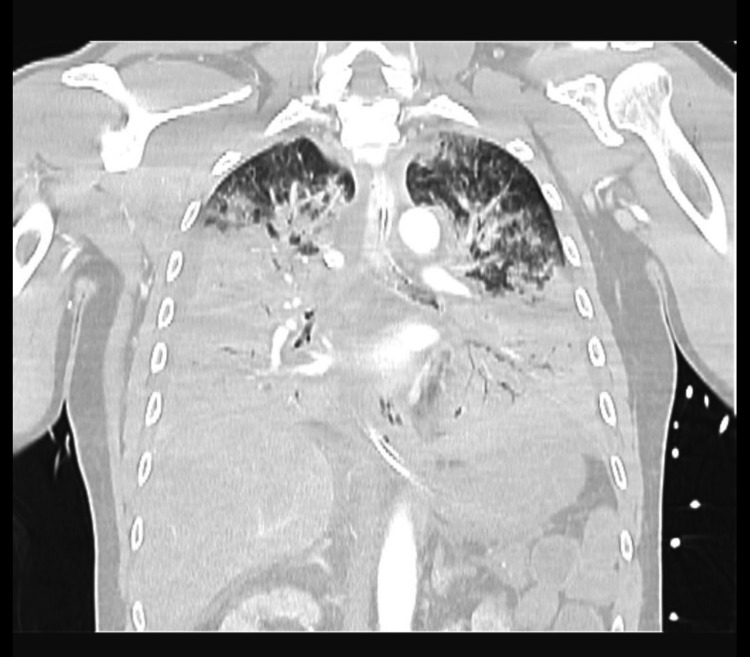
Thoracic-abdominopelvic CT on the second admission showing extensive lung consolidation bilaterally consistent with congestion

We report a case of a 58-year-old Caucasian male with a history of surgical splenectomy due to abdominal trauma at the age of 18. He had been previously hospitalised in July 2021 with pneumococcal shock with an unknown portal of entry; he was discharged after 18 days, making a full recovery. He had only done two doses of the 23-valent PPSV at that point (in 2007 and 2012), so he finished the immunisation schedule with the 13-valent PCV one month after being released from the hospital. After 11 months, he returned to the emergency department, reporting fever, shivering, myalgia, abdominal discomfort, and diarrhoea. Objectively, he was normotensive, slightly tachycardic, febrile, eupnoeic, did not need oxygen supply, and was without any other specific sign in physical examination. Laboratory tests showed a white blood cell (WBC) count of 12.120 x 10⁹/L, with 93% neutrophilia, C-reactive protein (CRP) 7.2 mg/L (reference range <0.3 mg/L), platelet count 239 x 10⁹/L, and serum creatinine (sCr) 1.36 mg/dL (Table 1). The chest X-ray was normal (Figure 1), and the arterial blood gas showed no signs of hyperlactatemia or respiratory failure. The pneumococcal urinary antigen test was positive, and he was discharged home and medicated with amoxicillin/clavulanic acid. After six hours, he returned to the emergency room in shock, with altered mental status, a very agitated, Glasgow Coma Scale score of 12 (E2 V4 M6), hypotensive, tachycardic, with evident signs of hypoperfusion, tachypnoeic with acute respiratory failure, and an exuberant erythematous rash (Figure 2). Laboratory tests revealed haemoconcentration with haemoglobin 21 g/dL, WBC 12 x 10⁹/L, and CRP 157 mg/L, aggravating acute kidney injury with sCr 3.60 mg/dL, severe thrombocytopenia with 28 x 10⁹/L, and hypoglycaemia (Table 1). Thoracic-abdominopelvic CT showed extensive lung consolidation bilaterally consistent with congestion, without any other alterations (Figure 3). A transthoracic echocardiogram confirmed ventricular hyperkinesia with small hypercontractile ventricles and a small inferior vena cava with marked respiratory variation without any other major alteration. He was intubated and ventilated, and he initiated fluid therapy with crystalloid and vasopressor support with norepinephrine (maximum 3.12 µg/kg/min). Due to the severity of the shock, hydrocortisone 200 mg and albumin 40 g were administered, and he started epinephrine. Regarding antibiotherapy, he has begun taking piperacillin, tazobactam, and vancomycin. Due to the exuberant presentation and the suspicion of streptococcal toxic shock, clindamycin 900 mg and intravenous immunoglobulin (IVIG) 1 g/kg (80 g) were also administered. Three fresh frozen plasmas, one platelet pool, and 10 mg of vitamin K were given to him, and he was put on continuous veno-venous haemofiltration. Despite all of these therapeutic interventions, he remained with refractory hypotension, respiratory failure with a PaO2/FiO2 ratio of 60, disseminated intravascular coagulation with uncontrollable bleeding from intravenous lines, catheters, and mucosal surfaces, and metabolic acidaemia. A real-time polymerase chain reaction assay detected the DNA of *S. pneumoniae* in blood and endotracheal aspirate. The detection for first line serotypes (3, 5, 7, 9, 14, 15, 16, 19, 20, 23, 33, 38) was negative. The sample volume was not enough to test for the second line serotypes (7C, 8, 10, 11, 12, 15A, 17F, 18, 19F, 22F, 31, 34, 35B, 35F).

**Table 1:** Comparison of laboratory values aPTT: activated partial thromboplastin time; PT: prothrombin time

Parameters	Patient’s results		Reference range
	First admission	Second admission	
Haemoglobin	15.5 g/dL	21.0 g/dL	13.0-18.0 g/dL
Platelet count	239 x 10⁹/L	28 x 10⁹/L	150-400 x 10⁹/L
White blood count	1.12 x 10⁹/L	12.01 x 10⁹/L	4.00-11.00 x 10⁹/L
C-reactive protein	7.2 mg/L	157.0 mg/L	<3.0 mg/L
Urea	49 mg/dL	73 mg/dL	10-50 mg/dL
Serum creatinine	1.36 mg/dL	3.60 mg/dL	0.67-1.17 mg/dL
Lactate	1.87 mmol/L	17.49 mmol/L	<2 mmol/L
aPTT	23.6 seconds	>180 seconds	24.2-36.4 seconds
PT	13.3 seconds	37.9 seconds	10.1-14.2 seconds
Fibrinogen	331 mg/dL	48 mg/dL	180-350 mg/dL

## Discussion

The risk of infection, sepsis, and sepsis-related mortality appears to be approximately two to three times higher in asplenic patients when compared with the general population [5-6]. Patients with impaired splenic function are at risk for severe and overwhelming infections with encapsulated bacteria, bloodborne parasites, and other infections where the spleen plays an important role. This organ has an abundance of lymphoid tissue, including splenic macrophages that are responsible for the opsonisation and phagocytosis of encapsulated organisms such as *S. pneumoniae*, *Haemophilus influenzae *(*H. influenzae*), and *Neisseria meningitidis (N. meningitidis)*. The spleen is also a major site of early immunoglobulin M production, which is important in the acute clearance of pathogens from the bloodstream. The overall case-fatality rate for *S. pneumoniae* bacteraemia is about 20%, but it may be as high as 60% among patients with asplenia [6-7].

Reviewing the case, the symptoms reported by the patient, fever, myalgia, and diarrhoea, in a splenectomised patient were the clue to an underlying serious process. Management of fever in asplenic patients includes immediate empiric intravenous antibiotic administration. Ceftriaxone and cefotaxime are both active against most *S. pneumoniae, H. influenzae* type b, and *N. meningitidis* isolates; vancomycin should be added if there is a risk of beta-lactam-resistant *S. pneumoniae*. Because progression to septic shock and respiratory distress can occur rapidly, preparations for fluid resuscitation, vasopressor support, and airway management should be made [6]. IVIG is controversial in sepsis and not recommended for the general population. However, as IVIG has the potential to offset the immune deficits of splenectomised patients, it is reasonable to give IVIG to selected patients with sepsis who have impaired splenic function [7-8].

*S. pneumoniae* has long been one of the most prominent bacterial causes of disease in humans and was one of the first to be identified as a cause of human infection. In spite of the widespread use of vaccines for more than 20 years, this disease is still responsible for approximately 1.6 million deaths worldwide each year [1].

In Portugal, epidemiological studies have documented a dynamic evolution in the serotypes, with an increase in cases of invasive pneumococci disease by serotypes not included in the PCV13. Most of them, however, are included in the PPSV23. The most common serotypes in Portugal from 2015 to 2018 were eight (19%), three (15%), 22F (7%), 14 (6%), and 19A (5%). As such, during this period, PCV13 covered 44% of the circulating serotypes, and PPSV23 covered 80%. In the population of Northern Portugal, 22F is the most common serotype not covered by PPSV23 or PCV13 [9].

During the summer of 2021, the United States Food and Drug Administration licensed two new PCVs for use in adults: PCV15 and PCV20. These vaccines target common serotypes causing invasive pneumococcal disease and pneumococcal pneumonia in the United States. PCV15 contains all PCV13 serotypes (1, 3, 4, 5, 6A, 6B, 7F, 9V, 14, 18C, 19A, 19F, 23F) plus 22F and 33F. PCV20 contains all PCV15 serotypes plus 8, 10A, 11A, 12F, and 15B [10].

In this case, the patient already had two doses of PPSV23 (15 and 10 years ago) and one of PCV13 approximately 11 months before the fatal event. According to the new Centers for Disease Control and Prevention guidelines, the recommendation for adults 19 through 64 years old with immunocompromising conditions who have received PCV13 and two doses of PPSV23 is to give no additional pneumococcal vaccine or to give one dose of PCV20 at least five years after the last pneumococcal vaccine [11]. This patient had PCV13 about a year before, so no vaccine is recommended. Due to recent and dubious guidelines with multiple recommendation choices, the authors advise that, when available, local epidemiology should be addressed in order to cover the most common serotypes, especially in high-risk individuals.

Reviewing European epidemiology, the last report by the European Centre for Disease Prevention and Control dates to 2018. At that time, the proportion of the five most frequent serotypes of *S. pneumoniae* that caused invasive pneumococcal disease in adults aged 65 or older was three (14.7%), eight (14.0%), 19A (7.6%), 22F (7.4%), and 9N (5.4%). As such, the proportion of serotypes covered by the PCV13 vaccine was 29%, and the proportion covered by PPSV23 was 73%. As such, an effort must be made to upgrade epidemiological data, as the frequency of serotypes not covered by PCV13 or PPSV23 should trigger the implementation of the new vaccines [12].

## Conclusions

Fever in a patient with impaired splenic function is an emergency, and it should be promptly and adequately identified and managed as it may have a fulminant course.

Despite the success of PCVs, with the reduction in PCV-serotype nasopharyngeal colonisation rates in children, leading to herd immunity and reduced incidence of invasive disease, serotypes continue to change as vaccine serotypes disappear from the community and other non-vaccine serotypes take their place. These two population-level phenomena, indirect effects (or herd immunity) and the emergence of replacement strains, contribute to a circle difficult to interrupt. The much-awaited broadly serotype-independent vaccine may be the “holy grail” of pneumococcal vaccine development. Until then, local epidemiology should help tailor the vaccination scheme where the recommendations are dubious.

The report of cases like this demonstrates the need for continuous serotype surveillance and vaccine development, as even with vaccination and all the therapeutic measures, there are still lives that cannot be saved.
